# Solid-State Dewetting Dynamics of Amorphous Ge Thin Films on Silicon Dioxide Substrates

**DOI:** 10.3390/nano10122542

**Published:** 2020-12-17

**Authors:** Dimosthenis Toliopoulos, Alexey Fedorov, Sergio Bietti, Monica Bollani, Emiliano Bonera, Andrea Ballabio, Giovanni Isella, Mohammed Bouabdellaoui, Marco Abbarchi, Shiro Tsukamoto, Stefano Sanguinetti

**Affiliations:** 1 L-NESS and Dipartimento di Scienza dei Materiali, Università di Milano Bicocca, 20126 Milano, Italy; emiliano.bonera@unimib.it (E.B.); shiro.tsukamoto@unimib.it (S.T.); stefano.sanguinetti@unimib.it (S.S.); 2 L-NESS and CNR-IFN, 20133 Como, Italy; alexey.fedorov@polimi.it (A.F.); monica.bollani@ifn.cnr.it (M.B.); 3 L-NESS, Dipartimento di Fisica, Politecnico di Milano, 20133 Como, Italy; andrea.ballabio@polimi.it (A.B.); giovanni.isella@polimi.it (G.I.); 4 CNRS, Aix-Marseille Université, Centrale Marseille, IM2NP, UMR 7334, 13013 Marseille, France; mohammed.bouabdellaoui@im2np.fr (M.B.); marco.abbarchi@im2np.fr (M.A.)

**Keywords:** solid state dewetting, amorphous materials, Ge thin films

## Abstract

We report on the dewetting process, in a high vacuum environment, of amorphous Ge thin films on SiO_2_/Si (001). A detailed insight of the dewetting is obtained by in situ reflection high-energy electron diffraction and ex situ scanning electron microscopy. These characterizations show that the amorphous Ge films dewet into Ge crystalline nano-islands with dynamics dominated by crystallization of the amorphous material into crystalline nano-seeds and material transport at Ge islands. Surface energy minimization determines the dewetting process of crystalline Ge and controls the final stages of the process. At very high temperatures, coarsening of the island size distribution is observed.

## 1. Introduction

Germanium continuous thin films are in a metastable state of equilibrium. As such, they can be transformed into isolated nano-islands or more complex architec-tures by annealing at high temperature [1,2,3]. The metamorphosis of the crystalline thin film into nanocrystals is kinetically controlled by surface diffusion [4]. This is a spontaneous phenomenon occurring at the wafer scale and it offers the advantage to create semiconductor nano-islands organized in multiple configurations depending on the initial film thickness, annealing temperature and time [5].

Solid-state dewetting of monocrystalline ultra-thin silicon on insulator (SOI) and Ge on insulator (GOI) is a scalable fabrication technique that allows the fine tuning of single crystal nano-islands’ size, size dispersion and surface distribution [6,7,8]. In this system, the main mechanisms at play are surface energy minimization and surface diffusion [9,10]. Both Si [11] and Ge [12] on an insulator have been recently reported and exploited as dielectric Mie resonators. This is an emerging alternative to the wide-ly employed plasmonic particles for the enhancement of light–matter interaction and light management at visible and near infrared frequencies.

The use of SOI and GOI for the fabrication of nano-islands by dewetting has sev-eral drawbacks, due to the limitation of the system to planar wafers and simple geom-etries. In addition, in the case of Ge on the insulator, the preparation of the starting material is lengthy and costly. The possibility to obtain crystalline nano-islands, with the desired size and size distribution by dewetting thin amorphous group IV semicon-ductors films deposited on SiO2, could be of fundamental importance for the imple-mentation of such intriguing photonic technology into complicated geometries, pat-terned substrates and actual photonic circuits, not counting the sensible reduction in costs of the Ge/SiO_2_/Si starting material. Despite its relevance for applications in pho-tonics, the mechanism of island formation from amorphous Ge thin layers has not yet been properly addressed.

Here, we present a detailed analysis of the dewetting process of amorphous Ge deposited at low temperature by low-energy plasma-enhanced chemical vapor deposi-tion (LEPECVD) on a SiO_2_ layer on Si (001). We monitor the formation of poly-crystals via in-situ high-energy electron diffraction and, after annealing, via Raman spectroscopy. We show that, in addition to surface energy minimization, which drives the dewetting in crystalline layers [4], Ge crystal nucleation and accretion and Ge diffusion on the a-Ge surface are fundamental actors in the process of transformation of the amorphous lay-ers into crystalline nano-islands. At high temperatures, coarsening of the island dis-tribution is observed pointing to the presence of the Ostwald ripening effect taking place between the c-Ge islands [13].

## 2. Materials and Methods

A thin layer of Ge (15 nm) was deposited by an in-house LEPECVD system, on top of 1 μm of SiO_2_ previously obtained by thermal oxidation of a Si (001) substrate [14]. The a-Ge layer was grown at 100 °C with a growth rate of 0.25 nm/s. The wafer was diced into sample pieces with approximately 1 cm^2^ size and mounted on a molyblock holder. Six samples were annealed at increasing temperature, in the range T = 550–780 °C, for 2 h in an ultra-high vacuum chamber (see Table 1).

During annealing, the background pressure was about 5 × 10^−9^ torr. The dewetting experiment was monitored in situ by reflection high-energy electron diffraction (RHEED). The morphological characterization of the samples was performed ex situ by scanning electron microscope (Philips XL30 SFEG SEM) imaging. To confirm the crystallinity of the islands we used a micro-Raman setup in a single spectrometer configuration using a λ = 532 nm laser as excitation source. A 100× (0.90 numerical aperture) objective lens was used for excitation and collection in backscattering configuration. The power on the sample was less than 1 mW. The sample was mounted on a stage with a 0.1 µm lateral resolution movement.

## 3. Results

The sample series is designed to address the dependence on temperature of the dewetting process dynamics (samples S1–S6, see Table 1). We monitored, at fixed annealing time, the evolution of the morphology of a thin (15 nm) a-Ge film at increasing temperatures in the T = 550 °C to T = 780 °C range.

The a-Ge film did not show any significant difference between the pre-and post-annealing structure at T = 550 °C. Here, we addressed temperature dependence, focusing on the crystallization and agglomeration dynamics of Ge islands from an amorphous Ge layer. However, we observed that for a longer annealing time we can expect a complete dewetting also in the low annealing temperature range. As regards the merit of this observation, the work of M. Aouassa et al. can be considered [15].

**Figure 1:**
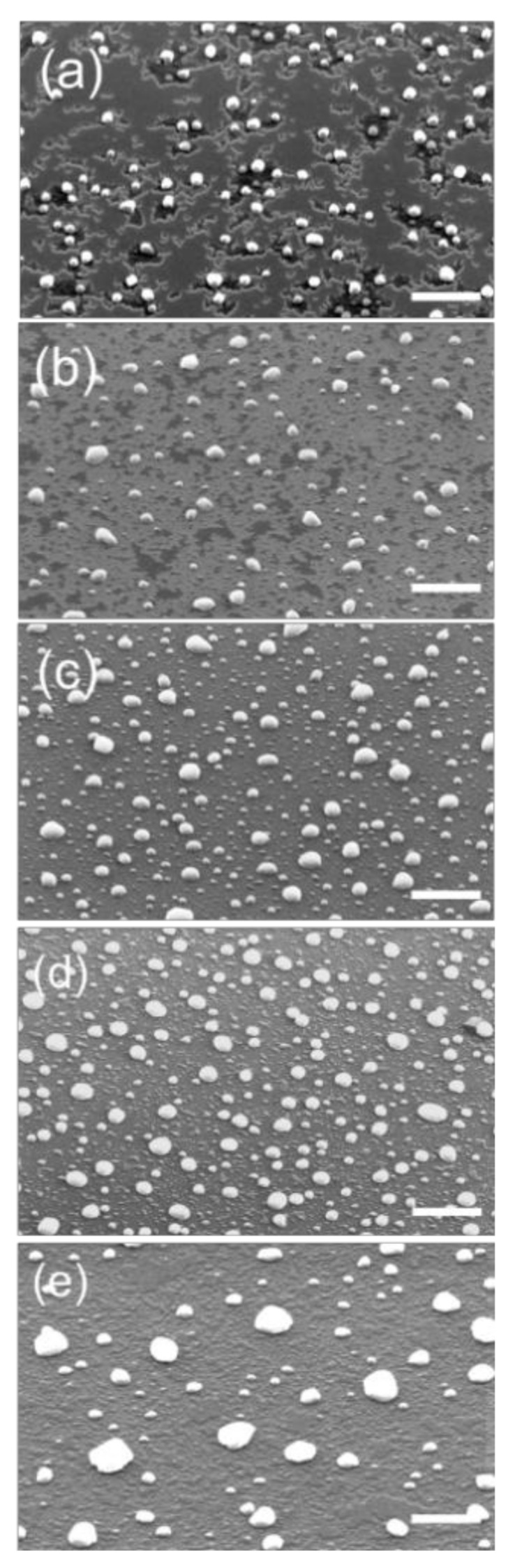
Scanning electron microscopy (SEM) images, measured in 45 degrees tilted view of samples S2 (**a**), S3 (**b**), S4 (**c**), S5 (**d**) and S6 (**e**). The white bar corresponds to the distance of 1 μm.

Increasing the temperature to T = 580 °C (Sample S2), the surface shows a substantial change (Figure 1a).

**Figure 2:**
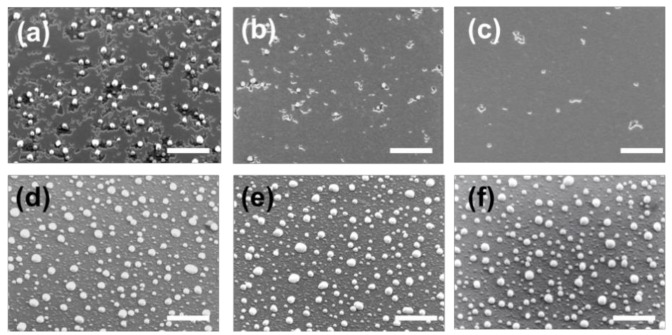
Upper line (**a**–**c**): SEM images taken in different spots of sample S2 (low annealing temperature T = 580 °C). Lower line (**d**–**f**): SEM images taken in different spots of sample S5 (high annealing temperature T = 750 °C). The white bar corresponds to 1 μm length.

Nano-islands appear on the surface, coexisting with the a-Ge film. The a-Ge film is still a 2D structure but characterized by the presence of trenches and openings, visible as dark areas in the SEM image, where the underlying SiO_2_ is exposed. The Ge islands show a density of ≈8 ± 1 × 10^8^ cm^−2^ and an average diameter D = 115 ± 30 nm (see Table 1). Dewetting initiates from openings and trenches on the surface of the thin film whose boundaries are characterized by dendritic shapes. Occasionally, the formation of islands is observed at the edge of the dewetted area. Openings and island formation are highly spatially non-uniform as shown in Figure 2a–c, where the SEM image from three different locations on the S2 sample, are reported. The presence of a high non-uniformity in crystallization behavior at 580 °C, which disappears at higher temperatures (Figure 2d–f), may be traced back to the presence of local variations in the film structure. In fact, during crystallization from the amorphous phase, an initial crystalline nucleus initially forms. The full development of a crystallite into a nanoscale c-Ge island takes place only after the crystallite reaches a minimum size at which the energy reduction associated with the formation of a crystal is greater than the energy increase associated with the formation of the interface between the c-Ge and the a-Ge phase [16]. Local variations in film structure, like thickness, surface morphology or the presence of defects or oxygen contamination induced by air exposure, are critical parameters for the crystallization process as they influence the free energy gain by crystallization, leading to local variations in the probability of stable c-Ge islands formation. Similar arguments have been used to explain the dependence of the crystallization temperature on the film thickness of a-Ge thin films [16]. In our case, this effect is enhanced by local temperature gradients, unavoidable in our experimental setup.

Obtaining more homogeneous samples in terms of dewetting stage and islands size distribution would be preferable especially when applications are concerned (such as Mie resonators for light management). In this respect, the use of larger substrates, higher temperature, or longer annealing time would improve these figures of merit.

For the following discussion, we focus on SEM images collected at the sample’s centers, minimizing edge effects. Increasing the temperature to T = 620 °C (Sample S3, Figure 1b), the a-Ge film loses connectivity, with huge material transfer toward the nano-islands. In sample S3 the island density is 25 ± 3 × 10^8^ cm^−2^ and the average diameter 73 ± 23 nm. At T = 680 °C (Sample S4, Figure 1c) the film becomes fully dewetted exposing the SiO_2_. Counter intuitively, the average diameter of the nano-islands decreases from D = 115 ± 30 nm at T = 580 °C (S2) to D = 55 ± 10 nm at T = 680 °C (S4), while the island density increases to 40 ± 2 × 10^8^ cm^−2^. The morphology observed in the fully dewetted sample S5 is uniform all over the sample surface, thus showing the stability of the process (Figure 2d–f) once the dewetting is obtained. Increasing further the annealing temperature to T = 750 °C (Sample S5, Figure 1d) the density of the larger islands does not change appreciably, while we observe a progressive disappearance of the smaller ones. In S5 the density of the island is 11 ± 1 × 10^8^ cm^−2^ and their average diameter 110 ± 56 nm. When the annealing temperature reaches T = 780 °C (Sample S6, Figure 1e), the smaller nano-islands are no longer present and we observe an increased coarsening of the size distribution of the larger ones, with an average diameter of 120 ± 102 nm and a density of 9 ± 1 × 10^8^ cm^−2^.

**Figure 3:**
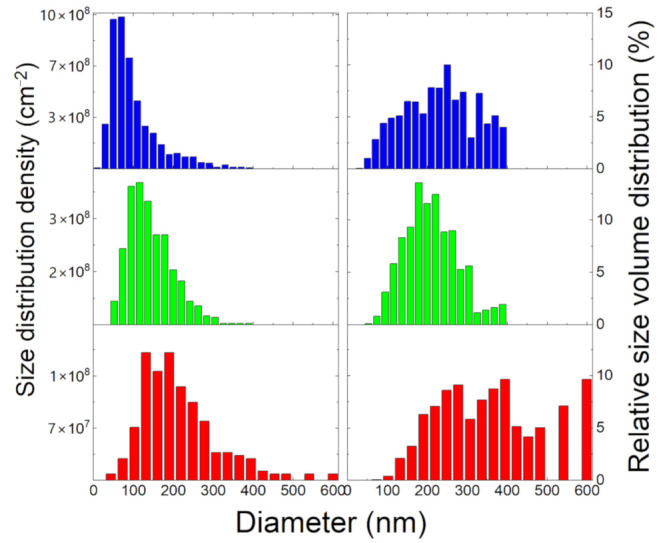
Left panels: size distribution density of sample S4 (**top**), S5 (**center**) and S6 (**bottom**). Right Panels: size distribution of the relative volume (in percent) of sample S4 (**top**), S5 (**center**) and S6 (**bottom**).

We carefully analyzed the size density distribution of the nano-islands in samples S4–S6 (Figure 3). These samples correspond to the configuration of full dewetting, where no residual patches of a-Ge are left on the SiO_2_ surface.

The size distribution in sample S4 shows an asymmetrical monomodal distribution peaked around 50 nm. The presence of many small islands is responsible for the steep increase of the island density between samples S3 and S4. Increasing the temperature to T = 750 °C (sample S5) the small islands completely disappear. Consequently, the peak of the distribution moves to larger sizes, at about 100 nm. A further increase of annealing temperature (T = 780 °C—sample S6) further moves the size distribution to larger values and adds a tail on the large island side.

**Figure 4:**
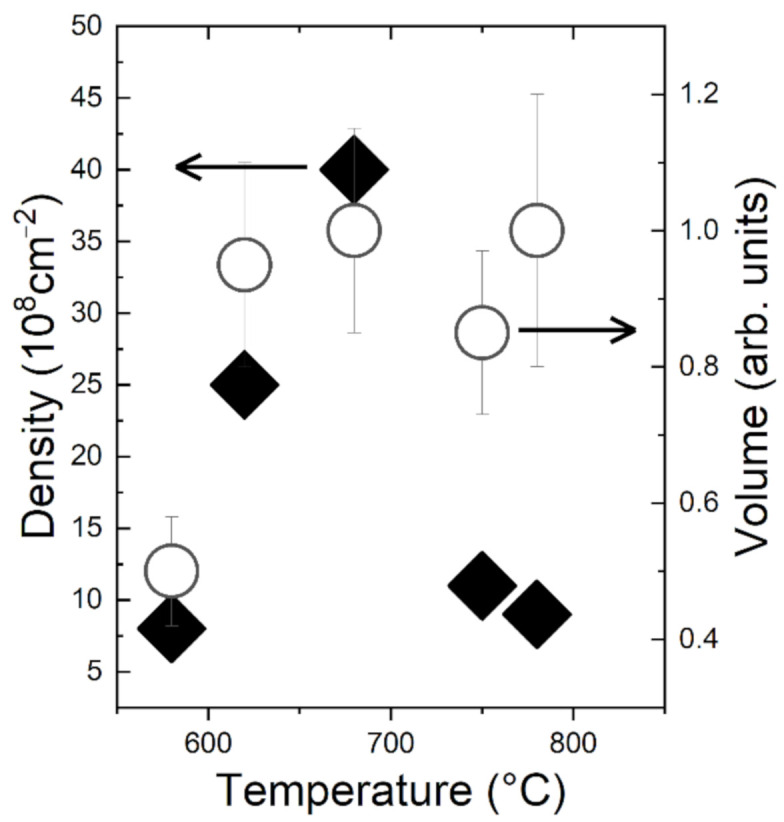
Temperature dependence of the ensemble island density (black diamonds) and ensemble island volume (white circles). The island volume is estimated through the formula V_ISL_ = ηd^3^, where d is the island diameter and η an undetermined constant which depends on the actual island shape.

Investigating the actual size dependence of the island volume distribution could add more depth to our observations. As the islands show a similar aspect ratio and shape (see Figure 3), the island volume can be estimated via V_ISL_ = ηd^3^, where d is the island diameter as estimated from the SEM image and η a constant which depends on the actual island shape. The island volume statistics are reported in Figure 3. On the low temperature side (sample S4 and S5), the effect of the increasing temperature is the reduction of the broadening of the volume distribution (sample S5). Further increasing the temperature leads to a clearly visible coarsening of the volume size distribution. At T = 780 °C (Figure 3) the larger islands (diameter > 400 nm) contain the larger part of the dewetted material. Additional insight can be gained through the comparison of the dependencies on the annealing temperature of the ensemble island density and the ensemble island volume (Figure 4). The island density clearly shows a peak in the density around 680 °C which corresponds to the full crystallization of the a-Ge into crystalline islands. Further increasing the temperature, while decreasing the density leaves unchanged the total island volume. When connected with the observed coarsening of the island size distribution, this is the fingerprint of Ostwald ripening, a strong mass exchange taking place between already formed crystalline islands [13].

**Figure 5:**
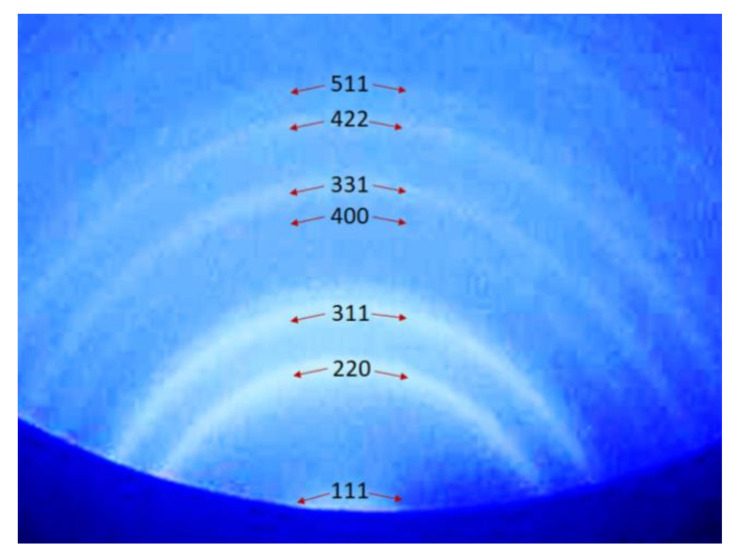
Reflection high-energy electron diffraction (RHEED) pattern measured on sample in annealed temperature T = 580 °C. The Bragg plane of the Ge lattice, corresponding to each Debye ring, is indicated.

In order to assess the crystallinity of the islands, we monitored by RHEED, the lattice characteristics of the film during the whole annealing process. Diffuse RHHED halo was observed in the low temperature range (T < 550 °C), in agreement with the presence of a-Ge. We observed the appearance of Debye rings at temperature T > 550 °C (Figure 5).

The appearance of Debye rings signals the presence of crystals on the surface, even if they have a random orientation with respect to each other. Such temperature coincides with the appearance of the nano-islands in the SEM images. The diffraction rings are attributed to the Ge crystal (511), (422), (331), (400), (311), (220) and (111) planes according to the literature [17], showing the crystalline nature of Ge nano-islands.

**Figure 6:**
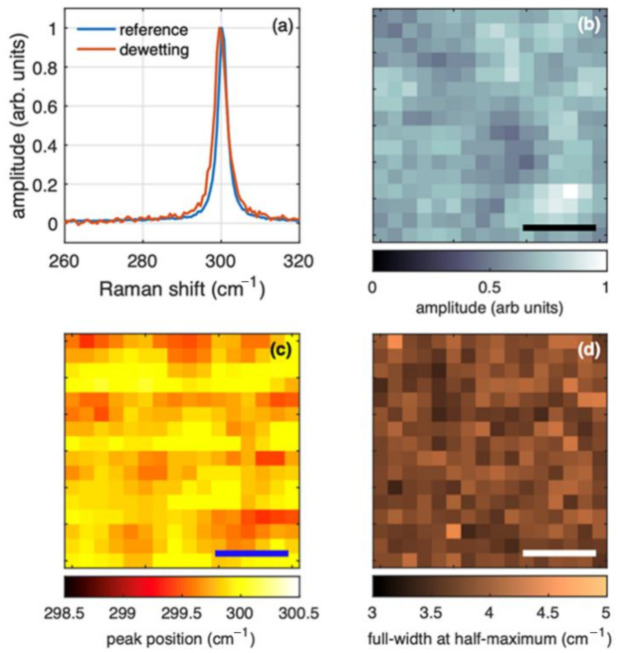
Raman spectroscopy of sample S6 (dewetting by annealing at 780 °C). (**a**) Comparison of normalized spectra from the dewetted germanium islands and a reference bulk germanium sample. (**b**–**d**) Spectral features from a Raman map collected from a 3 μm × 3 μm region with a sampling grid of 0.2 µm. (**b**) Amplitude. (**c**) Peak position. (**d**) Full width at half maximum. The thick mark in (**b**–**d**) is 1 µm wide.

Raman spectroscopy can provide further information about the morphology and structure of the sample. Figure 6 shows the results of a Raman mapping of sample S6, where according to the SEM images the dewetting is complete. The typical spectrum collected from the annealed sample is shown in panel (a) of Figure 6, superimposed to a reference bulk Ge for comparison.

The two spectra are nearly coinciding and there is no signal from amorphous germanium, which typically is between 280 and 290 cm^−1^ [18]. The very small spectral shift towards lower frequency for the sample obtained by dewetting with respect to the bulk crystalline Ge we took as reference sample, is a result of the small residual strain of the order of 0.1% [19] which can be attributed to a residual strain from the mismatch of the thermal expansion coefficient between Ge and SiO_2_. Panels (b) to (d) of Figure 6 show the result of the curve fitting of a Raman mapping collected from a 3 × 3 µm^2^ region of sample S6. The fluctuation of the amplitude of the Raman bands as a function of the position in panel (b) of Figure 6 are a result of the motion of the focus on larger and smaller islands, consistently with the SEM results in Figure 1e. Panel (c) and (d) of Figure 6 show, respectively, the corresponding peak position and FWHM, which are nearly constant through the investigated region. The extremely small fluctuations in the peak position can be interpreted easily in terms of different strain from different islands [20,21].

## 4. Discussion

Solid state dewetting of nanometer-scale films is a process by which a metastable film uncovers the substrate and accumulates into three-dimensional crystalline islands. The phenomenology of a-Ge is, therefore, clearly ascribable to this class of processes. However, models based on surface diffusion, driven by surface energy minimization, predict that, during dewetting, the film material is removed from the substrate and it accumulates in the rim of the receding film [22,23]. Eventually, the film breaks and leaves crystalline islands in front of a new dewetting front [11]. The evolution with the annealing temperature of surface morphology of metastable a-Ge samples into crystalline Ge islands shows marked differences with respect to the c-Ge film dewetting [24]. Comparing the dewetting dynamics for similar thickness of crystalline SiGe [3,12,25], crystalline Ge [24] with that of amorphous Ge [9], we observe that for this latter case the temperature range for obtaining well separated islands is in the 600 °C range against 700 °C or more for the former cases.

We observe the beginning of crystallization and dewetting a-Ge at about T = 580 °C. The crystallization temperature is therefore 80 °C higher than the well-established crystallization temperature of a-Ge T_cry_ = 500 °C [26]. However, both lower [9,13] (up to 275 °C) and higher [12] crystallization temperatures are reported in the literature. The great variability is attributed to sample preparation, layer thicknesses, interfaces, oxygen contamination and other extrinsic effects. In particular, the high temperature for the initiation of crystallization and dehumidification of the thin layers of a-Ge has been attributed to the presence of oxygen contamination on the surface [25]. Recent results by Camara et al. [27] confirm this interpretation. Crystallite formation in a-Ge nanowires occurred at a higher annealing temperature in thin nanowires than in thicker ones. Only nanowires with a diameter greater than 55 nm show a crystallization temperature of 500 °C, in agreement with that of bulk germanium [26]. The proposed interpretation is that oxygen atoms hinder both the formation and growth of crystallites by penetrating the nanowire more than the typical thickness of the native oxide, of the order of a few nm [28,29], thus interfering deeply with the crystallization dynamics in thin nanowires. The thickness of native oxide is typically not very stable under thermal treatments, being easily removed during annealing. The results by Camara et al. [27] thus show that native oxide removal may not solve the problem induced by oxygen contamination. For this reason, we prefer to skip the ex-situ chemical cleaning step. For the sake of thoroughness, we mention that for realistic applications of solid state dewetting of germanium (e.g., in photonics), is preferable to avoid the use of polluting and dangerous acids such as HF, and replace the chemical cleaning step with annealing.

Sample S2 (see Figure 1a) shows that the dewetting happens with the coexistence of nano-islands and a-Ge film in the same area, on the contrary of spinodal dewetting, where the nano-islands are formed at the retreating film boundary. The large spread in particles’ size is quantified by the standard deviation of the corresponding statistical distribution. These large values are typically found for this spontaneous process in semiconductors [26], and can be improved by templated dewetting [5,6,7] where very narrow size distributions are observed. The a-Ge film areas are characterized by trenches exposing the underlying SiO_2_ substrate. It is worth noting the dendritic shape of the trenches boundaries in the a-Ge film. The roughening of the a-Ge boundaries is in clear contrast with what is observed in crystalline film dewetting. There, the dewetting dynamics is determined by the reduction of the surface energies [30] and it is thus in contrast with the increase of the Ge–vacuum interface caused by the dendritic shape of the trenches.

From the analysis of the evolution with the annealing temperature of the metastable a-Ge film into crystalline islands we can, therefore, conclude that it is the outcome of two concurring phenomena that destabilize the film: (i) nucleation of crystalline seeds in the a-Ge film and (ii) surface energy minimization. In the dewetting process of c-Ge, the latter is the only driving force determining the overall dynamics. On the contrary, in the case of a-Ge, surface energy, while still in play, it shows a minor role in the evolution of the a-Ge film with the annealing temperature. This is shown by the dendritic shape of the trenches with their close-to-fractal geometry, which implies an increase of the exposed Ge surface. Still, surface energy plays a role in shaping the evolution of the c-Ge nano-island, which shows a compact 3D shape that minimizes the surface energy cost. The whole process is therefore determined by the nucleation of crystalline seeds in the a-Ge and the subsequent increase in size due to mass transport from the destabilized 2D film. Despite the central role of crystallization during annealing for dewetting, this point was not thoroughly addressed in past reports on the same topic [9,25].

On the low annealing temperature side, until the temperature reaches a critical value (in our case T > 550 °C), only a few crystalline nuclei are formed. These c-Ge seeds become the attraction point for the diffusing Ge atoms, increasing in size and evolving into nano-islands. The small number of islands acting as attraction site for the Ge diffusion may originate from the inhomogeneities in the film structure which may promote the local stability of critical nuclei for the island formation.

At higher annealing temperatures, the probability of nucleating a crystalline seed increases, thus increasing the density of the nano-islands as observed. The Ge mass transfer by diffusion from the a-Ge film to the c-Ge islands controls the actual kinetics of the film dewetting. As in the case of c-Ge dewetting, Ge diffusion proceeds on top of the Ge film [31]. We expect this process to proceed until a Ge bridge is maintained between the island and the a-Ge reservoir. Therefore, isolated islands are not growing in size and, conversely, isolated a-Ge patches can survive, if isolated from a nano-island, until a crystalline nucleus is formed within its boundaries. In agreement with this growth model, in sample S3 we observe the presence of a large number of small islands and the presence of a-Ge patches with no islands in close contact. The same process is responsible for the increased density and lower average size of the islands in Sample S4. Here, the dewetting is complete and the a-Ge film is fully transformed into nano-islands.

Increasing the temperature above this point decreases the nano-island density, leading to the progressive disappearance of the small nano-islands and the coarsening of the size distribution accompanied by a large mass transfer toward the larger islands. This phenomenology can be clearly observed in Figure 3. Increasing the temperature above 750 °C (sample S5) increases the average island size, making the small islands, which dominate sample S4 distribution, completely disappear. An increase of the temperature to 780 °C makes the sizable volume of the initial a-Ge film concentrate in a small number of large islands (see Figure 3). This is the typical effect of the Ostwald ripening among the nano-islands, which is activated by Ge atom detachment from the nano-islands and diffusion on the SiO_2_ substrate. As the temperature increases, the effect becomes stronger leading to an extremely coarsened size distribution.

## 5. Conclusions

Summarizing, we studied Ge nano-islands formation via solid state dewetting of amorphous Ge at annealing temperature below the Ge melting point. We used an inexpensive a-Ge thin film deposited on SiO_2_ by LEPECVD at low temperature (100 °C). The nano-islands formed are crystalline. The a-Ge film dewetting dynamics, while resulting in a similar array of isolated nano-islands as in the c-Ge case [1,3,5], is subjected to an additional driving force: the energy gain due to the crystallization process. The crystalline seeds act as effective collectors of diffusing Ge atoms, evolving into nanometer-size islands. The gain in energy due to the change of a-Ge into the crystalline phase is so relevant to overcome other sources of energy minimization, as shown by the film breakdown due to the development of dendritic trenches. Surface tension plays a role only in determining the final shape of the nano-islands, which show a compact 3D shape that can be related to surface energy minimization. Two temperature-activated mass-transfers are active during the process. The first, which dominates at intermediate temperatures (T > 550 °C), is the diffusion of Ge atoms from the a-Ge to the crystalline nuclei. The second, which determines the island size distribution at high temperatures, is Ostwald ripening.

## Figures and Tables

**Table 1:** a-Ge annealing temperature and nano-island characteristics (diameter and density) of the studied samples. The pristine a-Ge thickness was 15 nm.

Sample	Temp. (°C)	Average Diameter (nm)	Density (10^8^ Island/cm^2^)
S1	550	No dewetting	No dewetting
S2	580	115 ± 30	8 ± 1
S3	620	73 ± 23	25 ± 3
S4	680	55 ± 10	40 ± 2
S5	750	110 ± 56	11 ± 1
S6	780	120 ± 102	9 ± 1
